# Molecular mechanism analyses of post‐traumatic epilepsy and hereditary epilepsy based on 10× single‐cell transcriptome sequencing technology

**DOI:** 10.1111/cns.14702

**Published:** 2024-04-04

**Authors:** Fang Wen, Zhigang Tan, Dezhi Huang, Jun Xiang

**Affiliations:** ^1^ Department of Neurology The Second Xiang‐Ya Hospital of Central South University Changsha Hunan China; ^2^ Department of Neurosurgery The Second Xiang‐Ya Hospital of Central South University Changsha Hunan China

**Keywords:** DEGs, epilepsy, IL‐17 signaling pathway, single‐cell RNA sequencing, traumatic temporal lobe epilepsy

## Abstract

**Background:**

Single‐cell RNA sequencing analysis has been usually conducted on post‐traumatic epilepsy (PET) and hereditary epilepsy (HE) patients; however, the transcriptome of patients with traumatic temporal lobe epilepsy has rarely been studied.

**Materials and Methods:**

Hippocampus tissues isolated from one patient with PTE and one patient with HE were used in the present study. Single cell isolates were prepared and captured using a 10× Genomics Chromium Single‐Cell 3′ kit (V3) according to the manufacturer's instructions. The libraries were sequenced on an Illumina NovaSeq 6000 sequencing system. Raw data were processed, and the cells were filtered and classified using the Seurat R package. Uniform Manifold Approximation and Projection was used for visualization. Differentially expressed genes (DEGs) were identified based on a *p*‐value ≤0.01 and log fold change (FC) ≥0.25. Gene Ontology (GO, http://geneontology.org/) and KEGG (Kyoto Encyclopedia of Genes and Genomes, www.genome.jp/kegg) analyses were performed on the DEGs for enrichment analysis.

**Results:**

The reads obtained from the 10× genomic platform for PTE and HE were 39.56 M and 30.08 M, respectively. The Q30 score of the RNA reads was >91.6%. After filtering, 7479 PTE cells and 9357 HE cells remained for further study. More than 96.4% of the reads were mapped to GRCh38/GRCm38. The cells were differentially distributed in two groups, with higher numbers of oligodendrocytes (6522 vs. 2532) and astrocytes (133 vs. 52), and lower numbers of microglial cells (2242 vs. 3811), and neurons (3 vs. 203) present in the HE group than in the PTE group. The DEGs in four cell clusters were identified, with 25 being in oligodendrocytes (13 upregulated and 12 downregulated), 87 in microglia cells (42 upregulated and 45 downregulated), 222 in astrocytes (115 upregulated and 107 downregulated), and 393 in neurons (305 upregulated and 88 downregulated). The genes *MTND1P23* (downregulated), *XIST* (downregulated), and *RPS4Y1* (upregulated) were commonly expressed in all four cell clusters. The DEGs in microglial cells and astrocytes were enriched in the IL‐17 signaling pathway.

**Conclusion:**

Our study explored differences in cells found in a patient with PE compared to a patient with HE, and the transcriptome in the different cells was analyzed for the first time. Studying inflammatory and immune functions might be the best approach for investigating traumatic temporal lobe epilepsy in neurons.

## Graphical abstract

The reads obtained from 10× genomic platform of traumatic epilepsy and hereditary epilepsy were 39.56 M and 30.08 M. The Q30 of RNA reads over 91.6%. After filtering, 7479 traumatic epilepsy and 9357 hereditary epilepsy cells were left for further study. Over 96.4% of the reads are mapped to GRCh38/GRCm38. The cells were differentially distributed in two groups, with higher oligodendrocytes (6522 vs. 2532) and astrocytes (133 vs. 52), lower microglia (2242 vs. 3811) and neurons (3 vs. 203) were observed in hereditary epilepsy than traumatic epilepsy. The DEGs in four cell clusters were identified, with 25 in oligodendrocytes (13 upregulated and 12 downregulated), 87 in microglia (42 upregulated and 45 downregulated), 222 in astrocytes (115 upregulated and 107 downregulated), and 393 in neurons (305 upregulated and 88 downregulated). *MTND1P23* (downregulated), *XIST* (downregulated), and *RPS4Y1* (upregulated) were commonly expressed in four cell clusters. The DEGs in microglia and astrocytes were enriched in IL‐17 signaling pathway. Our study explored the cell grouping between traumatic and hereditary epilepsy, and the transcriptome in different cells for the first time. Inflammatory and immune functions might be the main approaches in traumatic temporal lobe epilepsy of neurons.

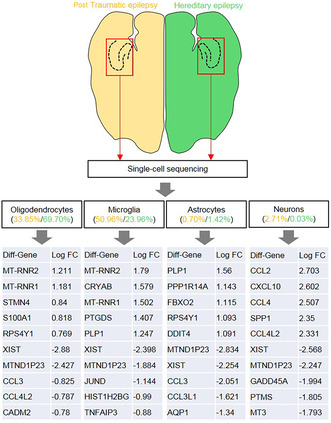

## 1 INTRODUCTION

Epilepsy is a chronic recurrent transient brain dysfunction syndrome that affects >70 million people worldwide,^1^ with the highest risk being in infants and older age groups. In China, the number of epilepsy patients reached 10 million in 2021.^2^ Epilepsy is divided into categories of post‐traumatic epilepsy (PTE) and hereditary epilepsy (HE) based on its causes. PTE refers to seizures that occur 1 week after a traumatic brain injury and have no obvious inducement. PTE accounts for 10%–20% of secondary epilepsy and 5% of all epilepsy.^3^ PTE seriously affects a patient's quality of life. The pathogenesis of PTE includes mechanical damage to the craniocerebral brain, oxidative stress, damage to the blood–brain barrier, peripheral immune response, and activation of thrombin.^4^ Anti‐epileptic drugs and surgery are the main treatments for PTE. However, the number of PTE patients who are resistant to anti‐epilepsy drugs is disappointing. As for HE, its main pathogenesis includes abnormalities of channel function, neurotransmitters, and neuroplastic cells.^5^ Genetic epilepsy may be related to mutations that occur in specific genes, such as sodium voltage‐gated channel alpha subunit 1 (*SCN1A*), potassium voltage‐gated channel subfamily A member 2 (*KCNA2*), potassium sodium‐activated channel subfamily T member 1 (*KCNT1*), and cyclin‐dependent kinase‐like 5 (*CDKL5*). The treatments for HE vary, and include etiology treatment, traditional drugs, and novel anti‐epileptic drugs. While the pathogenic links between PTE and HE seem to be clear, one study demonstrated that certain genetic factors increased the occurrence of PTE,^6^ indicating that genetic studies might be needed to further explore PTE pathology and produce better methods for treating PTE. Therefore, an investigation of differences or associations between PTE and HE is in highly meaningful. In recent years, many studies have attempted to explore the specific pathogenic mechanisms of PTE and HE. However, reports on differences between the molecular mechanisms of PTE and HE are still lacking.

Transcriptome analyses of epilepsy have been reported in several studies.^7^, ^8^, ^9^ Dong et al.^7^ described the relationship between inflammation‐related genes and cognitive dysfunction and epileptogenesis during drug administration for epilepsy, and they identified novel therapeutic targets for treating traumatic temporal lobe epilepsy (TLE) and its comorbidities. However, normal RNA‐Seq only shows the whole picture of the transcriptome and reflects an average gene expression profile across heterogeneous populations of cells.^10^ While the gene expression pattern usually differs in different cell types, a more accurate cell‐type‐specific transcriptome analysis is needed to gain a detailed understanding of cellular functions. The emergence of single‐cell RNA sequencing (scRNA‐Seq) technology has solved this problem and increased our knowledge concerning the global landscape of various diseases. ScRNA‐Seq has been used to analyze the pathogenesis of sepsis and revealed the importance of sustaining immune cell dysfunction in sepsis.^11^ ScRNA‐Seq is also employed in brain‐related disorder diseases, including autism spectrum disorder, in which the genes in neurons were found to correlate with clinical severity.^12^ Numerous scRNA‐Seq studies have been performed on epilepsy. For example, Tome‐Garcia et al.^13^ used scRMA‐Seq, cell‐type‐specific isolation, and transcriptomic profiling to investigate glial pathology in human traumatic temporal lobe epilepsy. Masuda et al.^14^ and Sankowski et al.^15^ analyzed the specific cellular functions of certain brain areas in epilepsy and examined the gene functions in microglial clusters. However, the former molecular mechanism studies usually focused on investigating epilepsy patients when compared to healthy individuals, and no study has focused on the difference between PTE and HE.

In our present study, we explored the dynamic transcriptome in various cells from one patient with PTE and one patient with HE. Furthermore, we also investigated the differentially expressed genes (DEG) implicated in the two types of epilepsy. This study is the first to use scRNA‐Seq to investigate differences in the molecular mechanisms of PTE versus HE. Our goal was to gain insights into cell type‐specific transcriptomic changes by performing unbiased scRNA‐Seq.

## 2 MATERIALS AND METHODS

### 2.1 Patients and tissue dissociation

One patient with PTE and one patient with HE were enrolled in the study. Both patients provided their signed written informed consent for study participation. This study was carried out in compliance with the Helsinki Declaration II and the Chinese Standards for Good Clinical Practice. The study protocol approved by the Ethics Committee of Second Xiang‐Ya Hospital of Central South University, Changsha, China (ethics review number 2020‐yan482).

Prior to the operation, baseline characteristics of both patients (age, gender, electroencephalography [EEG] results, imaging results, and pathological information) was collected and recorded. Next, samples of hippocampus tissue were collected from the epilepsy patients with congenital dysplastic and traumatic injuries. The tissues were immediately washed with calcium‐ and magnesium‐free PBS, and then stored in tissue protective fluid and sent to Hangzhou Lianchuan Biotechnology Co., Ltd (Hangzhou, China) for further use.

### 2.2 Preparation of single‐cell suspensions

Single‐cell suspensions were prepared for use in sequencing studies. Samples of hippocampus tissue were placed in a dissociation solution and shaken for 20 min at 100 rpm in a 37°C water bath. Tissue digestion was terminated by addition of PBS containing 10% fetal bovine serum (FBS, v/v). Next, the remaining cell suspension was filtered through a 70–30 μm strainer and centrifuged at 300 *g* for 5 min. The cells were then resuspended in PBS prior to incubation with red blood cell lysis buffer (MACS 130‐094‐183, ×10). After incubation, the suspension was centrifuged at 300 *g* for 5 min at 25°C, and the dead cells were removed using a Miltenyi^®^ Dead Cell Removal Kit (MACS 130‐090‐101). Finally, the cells were resuspended in PBS, observed using the trypan blue exclusion method, and counted with a hemocytometer/Countess II Automated Cell Counter. The overall cell viability was >85%, and the final cell concentration was adjusted to 700–1200 cells/μL.

### 2.3 Chromium 10× genomics library construction and sequencing

Approximately 5000 single cells were captured by using a 10× Genomics Chromium Single‐Cell 3′ kit (V3) according to the manufacturer's instructions. Next, a standard RNA library with a read length of 200–300 bp was constructed according to a standard protocol. The libraries were sequenced on an Illumina NovaSeq 6000 sequencing system (paired‐end, 150 bp) at a minimum depth of 20,000 reads per cell.

### 2.4 Raw data processing, cell filtering, and single‐cell subpopulation classification

The raw data were converted to the FASTQ format using Illumina bcl2fastq software (Illumina, San Diego, CA, USA). Cell Ranger pipeline (version 4.0.0, https://support.10xgenomics.com/single‐cell‐geneexpression/software/pipelines/latest/what‐is‐cell‐ranger) was employed for sample demultiplexing, barcode processing, single‐cell 3′ gene counting, and genome aligning (GRCh38/GRCm38, v96).

Next, the Seurat R package (version 3.1.1)^16^ was used for quality control by removing genes expressed in fewer than three cells or expressed in >500 cells as low and <5000 as high, and depleting UMI counts <500; the percentage of mitochondrial‐DNA derived gene‐expression was <25%. The Seurat R package was also used for data filtering and normalization, principal component analysis (PCA), and t‐distributed Stochastic Neighbor Embedding (tSNE) with the thresholds previously reported.^16^

### 2.5 Marker gene differentiation and enrichment analysis

The Seurat R package was used to analyze the DEGs in different cell populations by the bimodal likelihood ratio statistical test and identify genes that were up‐regulated in >10% of cells in different cell populations according to the following criteria: *p* ≤ 0.01 and log Fold‐change (FC) ≥ 0.25. Gene Ontology (GO, http://geneontology.org/) and Kyoto Encyclopedia of Genes and Genomes (KEGG, www.genome.jp/kegg) analyses were performed on the DEGs for an enrichment analysis.

### 2.6 Statistical analysis

Values for variables with a normal distribution are expressed as a mean value ± standard deviation (SD), and were analyzed by the *t*‐test. A *p*‐value <0.05 was considered to be statistically significant. GraphPad Prism 10 (San Diego, CA, USA) was used for image drawing and the unpaired student's *t*‐test analysis.

## 3 RESULTS

### 3.1 Baseline characteristics of patients

To explore the dynamic transcriptomes of PTE and HE, samples of hippocampal tissue were surgically removed from two patients and subjected to scRNA‐Seq on a 10× Genomics platform. An 18‐year‐old man diagnosed with PTE with a medical history of sodium valproate (200 mg bid) and oxcarbazepine (300 mg bid) use was involved in our present study. This patient was injured in a car accident 2 years earlier, and suddenly started twitching his limbs, foaming at the mouth, became delirious, and was unable to call; these symptoms lasted for approximately 3 min. A craniotomy exploration and hematoma removal were performed on the patient, but the PTE symptoms continued to occur once a month. The other patient in this study was a 15‐year‐old teenager who had been diagnosed with HE. When this patient was 3 years old, the first attack was occurred and was characterized by limb convulsions, foaming at the mouth, eyes upturned, and unconsciousness lasting approximately 30 min. The HE symptoms for this occurred once every 10 days, and he accepted lamotrigine (75 mg bid) treatment. Characteristics of the two patients are described in Table S1.

### 3.2 Raw data processing, cell filtering, and single‐cell subpopulation classification

The reads obtained from the 10× genomic platform of PTE and HE were 39.56 M and 30.08 M, respectively (Table 1). The Q30 score of the RNA reads was >91.6%, indicating the high quality of the reads for the following analysis. The other indexes of valid barcodes, sequencing saturation, Q30 bases in the barcode, and Q30 bases in UMI are listed in Table 1.

**TABLE 1:** The basic sequencing and mapping information.

Sample	TE	HE
Basic sequencing information		
Number of reads (M)	39.56	30.08
Valid barcodes	98.1%	98.3%
Sequencing saturation	67.2%	58.1%
Q30 bases in barcode	95.3%	95.4%
Q30 bases in RNA read	92.3%	91.6%
Q30 bases in UMI	92.5%	93.2%
Mapping information		
Estimated number of cells	7717	9918
Cells after filtering	7479	9357
Mean reads per cell	51,267	30,328
Median genes per cell	2052	2038
Reads mapped to genome	96.5%	96.4%
Reads mapped confidently to genome	92.8%	92.4%
Reads mapped confidently to intergenic regions	5.2%	3.7%
Reads mapped confidently to intronic regions	43.3%	35.4%
Reads mapped confidently to exonic regions	44.3%	53.3%
Reads mapped confidently to transcriptome	40.4%	48.9%
Reads mapped antisense to gene	1.8%	1.8%
Fraction reads in cells	92.4%	88.7%
Total genes detected	33,441	31,783
Genes after filtering	2068	2091

Abbreviations: HE, hereditary epilepsy sample; TE, traumatic epilepsy sample.

Totals of 7717 and 9918 cells were identified in the PTE and HE groups, respectively, which is enough for further cell classification (Table 1). After filtering, 7479 PTE cells and 9357 HE cells remained for further study. More than 96.4% of the reads were mapped to GRCh38/GRCm38, demonstrating uncontaminated and reliable data. Additional mapping information is shown in Table 1.

### 3.3 Cell grouping

**FIGURE 1:**
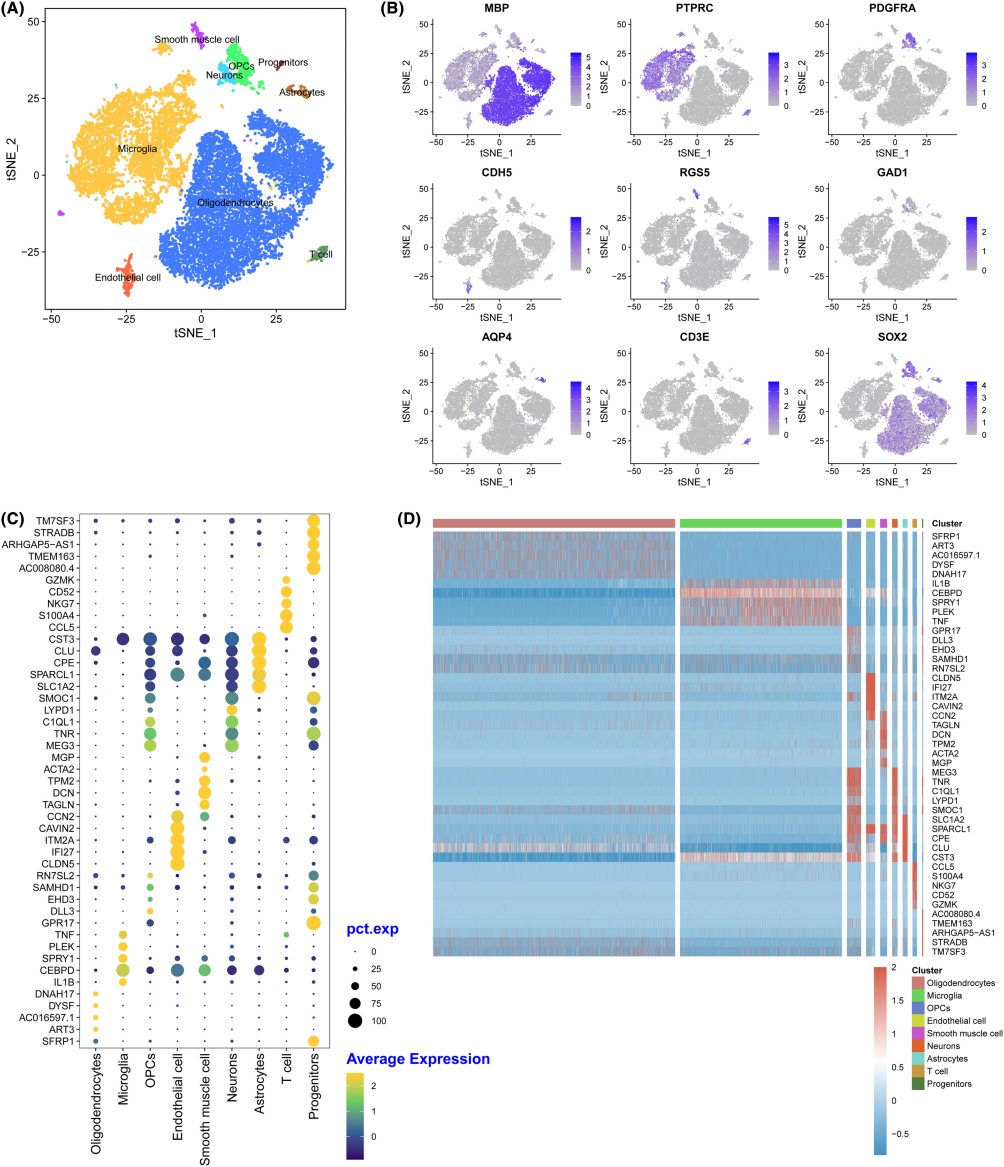
Cell clustering and marker gene identification. (A) t‐SNE clustering of snRNA‐Seq data. Cell types were annotated to the expression of known marker genes and exhibited by t‐SNE visualization with the same cells merged. (B) A bubble chart displaying the top five genes in each cluster. (C) The expression of classic marker genes displayed by a tSNE plot. Each dot represents a cell. The darker the dot, the higher the marker gene expression in a specific cell. (D) Heatmap of the marker genes in each cluster.

After stringent quality control, the remaining cells were annotated to different cell types with known marker genes. Cells were grouped into 10 clusters, including oligodendrocytes, microglial cells, OPCs, endothelial cells, smooth muscle cells, neurons, astrocytes, microglia, T cells, and progenitors (Figure 1A). In each cluster, genes with high specificity and expression (logFC >0.25) and expressed in at least 25% of all cells were identified as marker genes. All the marker genes identified are listed in Appendix S1. In addition, the top five genes identified in each cluster are shown in Figure 1B. Classic markers such as myelin basic protein (*MBP*), Protein Tyrosine Phosphatase Receptor Type C (*PTPRC*), and sex‐determining region Y‐box2 (*SOX2*) were also highly expressed in the annotated cells (Figure 1C). Figure 1D shows that different marker genes distinguished the various clusters. Oligodendrocytes were marked by secreted frizzled‐related protein 1 (*SFRP1*), ADP‐ribosyltransferase 3 (*ART3*), AC016597.1, dysferlin (*DYSF*), *DNAH17*, microglia marked interleukin 1 (*IL1B*), CCAAT/enhancer binding protein delta (*CEBPD*), Sprouty RTK signaling antagonist 1 (*SPRY1*), pleckstrin (*PLEK*), and tumor necrosis factor (*TNF*).

### 3.4 Clusters were differentially distributed in the two groups

**FIGURE 2:**
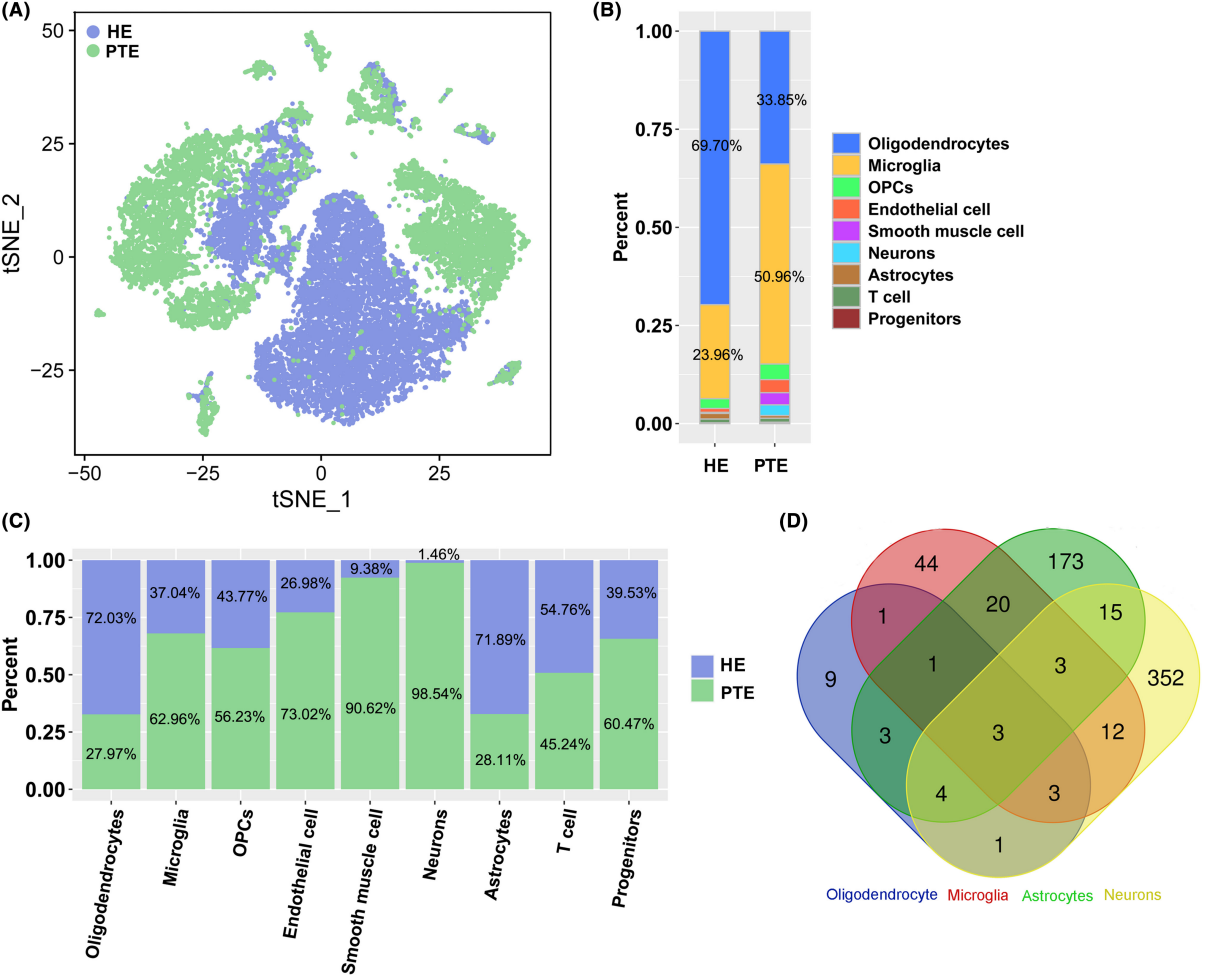
Cell clusters were differentially distributed in two groups. (A, B) show the number of cells by tSNE and a bar chart. (C) Bar chart showing the detailed ratio of cells in samples. (D) A Venn chart of the DEGs in four clusters. Three genes were co‐expressed in four clusters.

By comparing the distribution of different cell types in different groups, the differentially distributed cell types in a particular group were identified. We found that the cells were differentially distributed in the two groups (Figure 2A,B). The oligodendrocytes (69.70%) were dominant in HE and followed by microglial cells (23.96%). As for TE, microglia cells (50.96%) showed the highest percentage, followed by oligodendrocytes (33.85%). The detailed cell numbers and percentages in HE and PTE are listed in Table S2. The distribution of HE and PTE varied in a single cell (Figure 2C). Oligodendrocytes (72.03% vs. 27.97%), astrocytes (71.89% vs. 28.11%), and T cells (54.76% vs. 45.24%) were more predominant in HE than that in TE, while, microglia cells (62.96% vs. 37.04%), OPCs (56.23% vs. 43.77%), endothelial cells (73.02% vs. 26.98%), smooth muscle cells (90.62% vs. 9.38%), neurons (98.54% vs. 1.46%), and progenitors (60.47% vs. 39.53%) was more predominant in PTE than that in HE.

### 3.5 DEG identification in four clusters

The DEGs in four cell clusters were identified, with 25 in oligodendrocytes (13 upregulated and 12 downregulated), 87 in microglia (42 upregulated and 45 downregulated), 222 in astrocytes (115 upregulated and 107 downregulated), and 393 in neurons (305 upregulated and 88 downregulated) (Table S3). Among all the DEGs, only three genes were commonly expressed in all four cell clusters (Figure 2D), these included MT‐ND1 Pseudogene 23 (*MTND1P23*; downregulated), X inactivation‐specific transcript (*XIST*, downregulated), and 40S ribosomal protein S4 (*RPS4Y1*; upregulated).

### 3.6 DEGs identification and function analysis in oligodendrocytes

**FIGURE 3:**
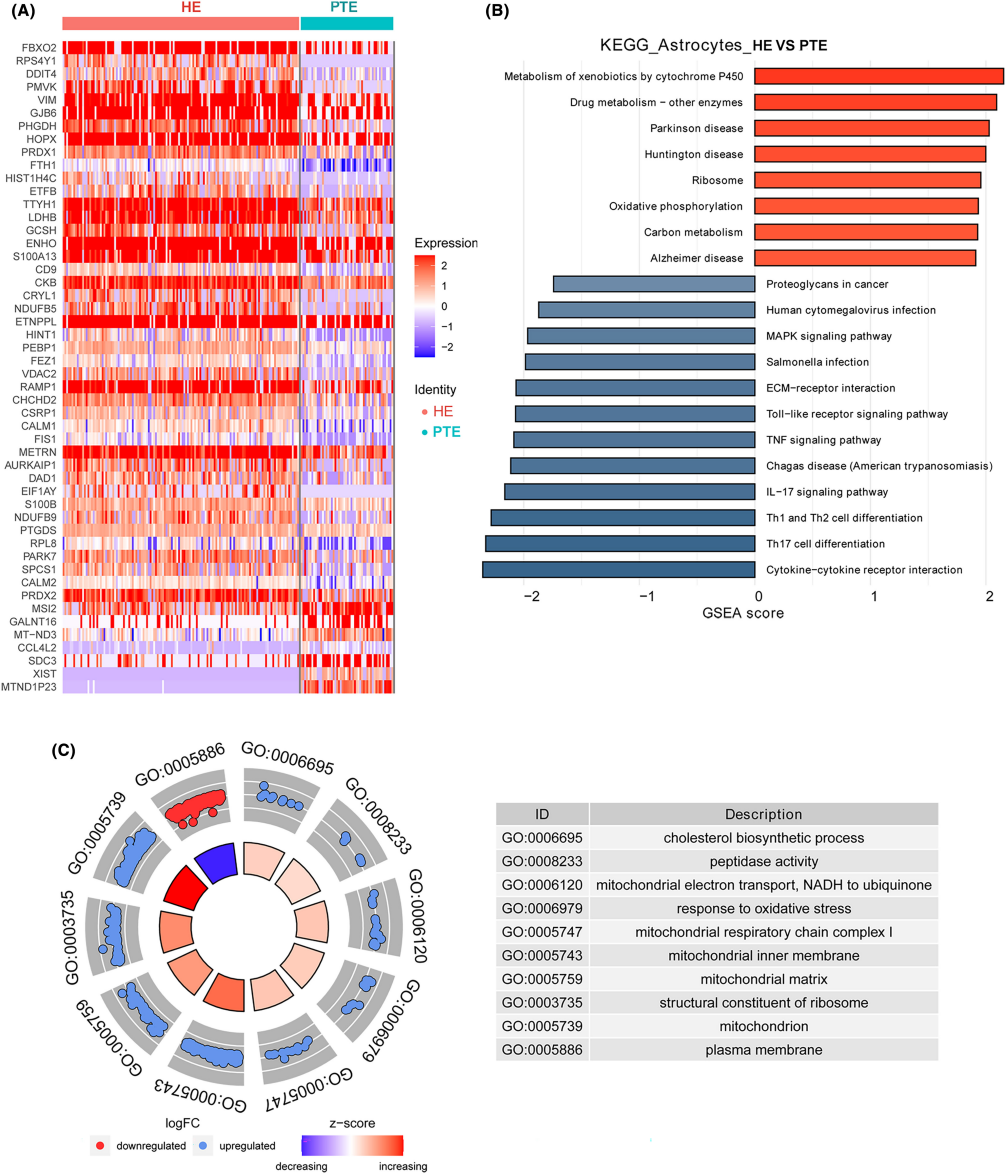
DEGs identification and function analysis in oligodendrocytes. (A) A heatmap of DEGs in oligodendrocytes. (B) The KEGG enrichment of DEGs in oligodendrocytes. (C) A GO analysis of DEGs in oligodendrocytes. DEGs, differentially expressed genes; GO, Gene Ontology; KEGG, Kyoto Encyclopedia of Genes and Genomes.

Only 25 DEGs were identified in oligodendrocytes, with 13 being upregulated and 12 downregulated (Appendix S2; Figure 3A). The most upregulated DEGs were *MT‐RNR2*, *MT‐RNR1*, stathmin‐like 4 (*STMN4*), *S100A1*, and *RPS4Y1*; the most downregulated DEGs were *XIST*, *MTND1P23*, CC chemokine ligands 3 (*CCL3*), C‐C motif chemokine ligand 4 like 2 (*CCL4L2*), and cell adhesion molecule 2 (*CADM2*) (Figure S1, Table 2).

**TABLE 2:** List of the most upregulated and downregulated genes in four clusters.

Oligodendrocyte		Microglia		Astrocytes		Neurons	
Gene name	Log FC	Gene name	Log FC	Gene name	Log FC	Gene name	Log FC
MT‐RNR2	1.211	MT‐RNR2	1.790	PLP1	1.560	CCL2	2.703
MT‐RNR1	1.181	CRYAB	1.579	PPP1R14A	1.143	CXCL10	2.602
STMN4	0.840	MT‐RNR1	1.502	FBXO2	1.115	CCL4	2.507
S100A1	0.818	PTGDS	1.407	RPS4Y1	1.093	SPP1	2.350
RPS4Y1	0.769	PLP1	1.247	DDIT4	1.091	CCL4L2	2.331
XIST	−2.880	XIST	−2.398	MTND1P23	−2.834	XIST	−2.568
MTND1P23	−2.427	MTND1P23	−1.884	XIST	−2.254	MTND1P23	−2.247
CCL3	−0.825	JUND	−1.144	CCL3	−2.051	GADD45A	−1.994
CCL4L2	−0.787	HIST1H2BG	−0.990	CCL3L1	−1.621	PTMS	−1.805
CADM2	−0.780	TNFAIP3	−0.880	AQP1	−1.340	MT3	−1.793

Abbreviation: FC, fold changes.

The top 10 KEGG pathways enriched in the DEGs of oligodendrocytes were chemical carcinogenesis, ribosome biogenesis in eukaryotes, glycolysis/gluconeogenesis, proteasome, fluid shear stress and atherosclerosis, carbon metabolism, retrograde endocannabinoid signaling, non‐alcoholic fatty liver disease (NAFLD), ribosome, and oxidative phosphorylation (Figure 3B).

The top 10 GO terms identified by DEGs in oligodendrocytes were DNA‐binding transcription factor activity, RNA polymerase II‐specific, protein phosphorylation, cell adhesion, G protein‐coupled receptor signaling pathway, mitochondrial respiratory chain complex IV, glutathione peroxidase activity, cytochrome‐c oxidase activity, gluconeogenesis, mitochondrial proton‐transporting ATP synthase complex, and mitochondrial ribosome (Figure 3C).

### 3.7 DEGs identification and function analysis in microglia

**FIGURE 4:**
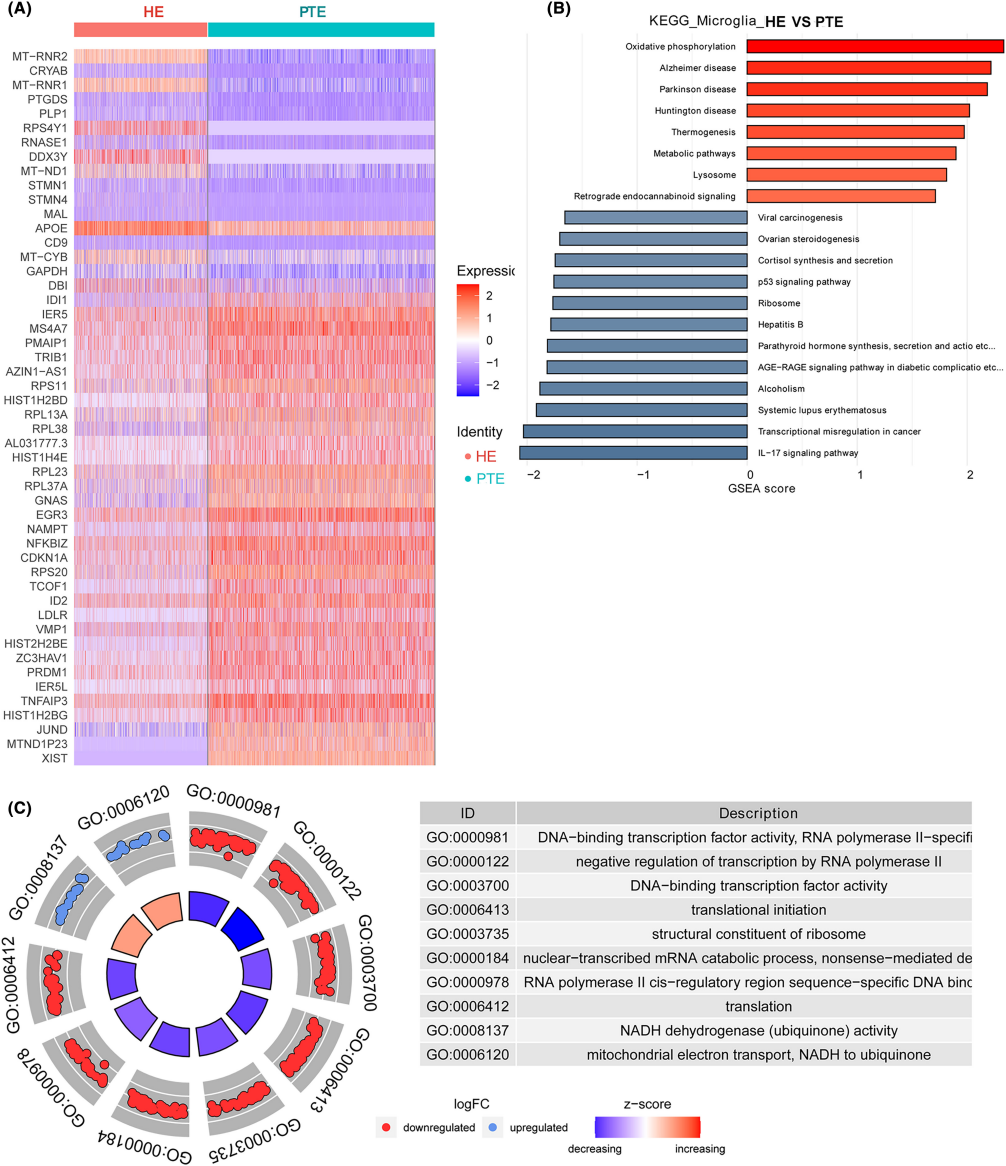
DEGs identification and function analysis in microglial cells. (A) A heatmap of DEGs in microglia cells. (B) The KEGG enrichment of DEGs in microglia cells. (C) A GO analysis of DEGs in microglial cells. DEGs, differentially expressed genes; GO, Gene Ontology; KEGG, Kyoto Encyclopedia of Genes and Genomes.

A total of 87 DEGs were identified in microglia cells, with 42 being upregulated and 45 downregulated (Appendix S3; Figure 4A). The most upregulated DEGs were *MT‐RNR2*, crystallin alpha‐B protein (*CRYAB*), MT‐RNR1, prostaglandin D synthase (*PTGDS*), and proteolipid protein 1 (*PLP1*); the most downregulated DEGs were *XIST*, *MTND1P23*, *JUND*, *HIST1H2BG*, and TNF‐inducible protein 3 (*TNFAIP3*) (Figure S2).

The top 10 KEGG pathways enriched in DEGs of microglia were IL‐17 signaling pathway, oxidative phosphorylation, Parkinson's disease, Alzheimer's disease, Huntington's disease, thermogenesis, transcriptional misregulation in cancer, metabolic pathways, systemic lupus erythematosus, and alcoholism (Figure 4B).

The top 10 GO terms identified by DEGs in microglial cells were DNA‐binding transcription factor activity, RNA polymerase II‐specific, negative regulation of transcription by RNA polymerase II, DNA‐binding transcription factor activity, translational initiation, structural constituent of ribosome, nuclear‐transcribed mRNA catabolic process, nonsense‐mediated decay, RNA polymerase II cis‐regulatory region sequence‐specific DNA binding, translation, NADH dehydrogenase (ubiquinone) activity, mitochondrial electron transport, and NADH to ubiquinone (Figure 4C).

### 3.8 DEGs identification and function analysis in astrocytes

**FIGURE 5:**
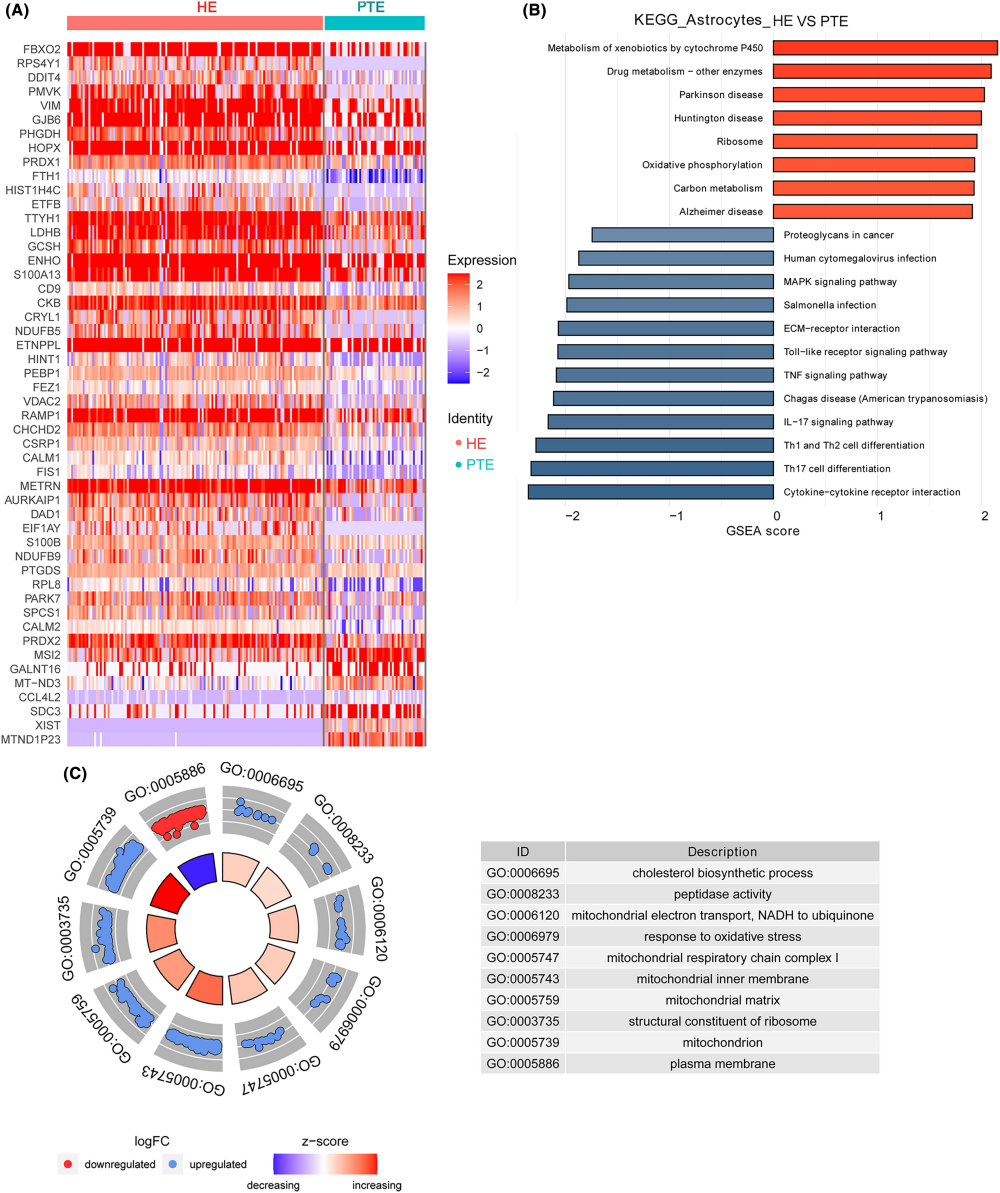
DEGs identification and function analysis in astrocytes. (A) A heatmap of DEGs in astrocytes. (B) The KEGG enrichment of DEGs in astrocytes. (C) A GO analysis of DEGs in astrocytes. DEGs, differentially expressed genes; GO, Gene Ontology; KEGG, Kyoto Encyclopedia of Genes and Genomes.

A total of 222 DEGs were identified in microglial cells, with 115 being upregulated and 107 downregulated (Appendix S4; Figure 5A). The most upregulated DEGs were *PLP1*, putative protein phosphatase‐1 inhibitory protein (*PPP1R14A*), F‐Box 2 (*FBXO2*), *RPS4Y1*, and DNA Damage Inducible Transcript 4 (*DDIT4*); the most downregulated DEGs were *MTND1P23*, *XIST*, *CCL3*, *CCL3L1*, and aquaporin 1 (*AQP1*) (Figure S3).

The top 10 KEGG pathways enriched in DEGs in astrocytes were MAPK signaling pathway, Human cytomegalovirus infection, Chagas disease (American trypanosomiasis), IL‐17 signaling pathway, Cytokine‐cytokine receptor interaction, Th17 cell differentiation, Th1 and Th2 cell differentiation, Toll‐like receptor signaling pathway, Metabolism of xenobiotics by cytochrome P450, and Drug metabolism—other enzymes (Figure 5B).

The top 10 GO terms identified in DEGs in microglial cells were cholesterol biosynthetic process, peptidase activity, mitochondrial electron transport, NADH to ubiquinone, response to oxidative stress, mitochondrial respiratory chain complex I, mitochondrial inner membrane, mitochondrial matrix, structural constituent of ribosome, mitochondrion, and plasma membrane (Figure 5C).

### 3.9 DEGs identification and function analysis in neurons

**FIGURE 6:**
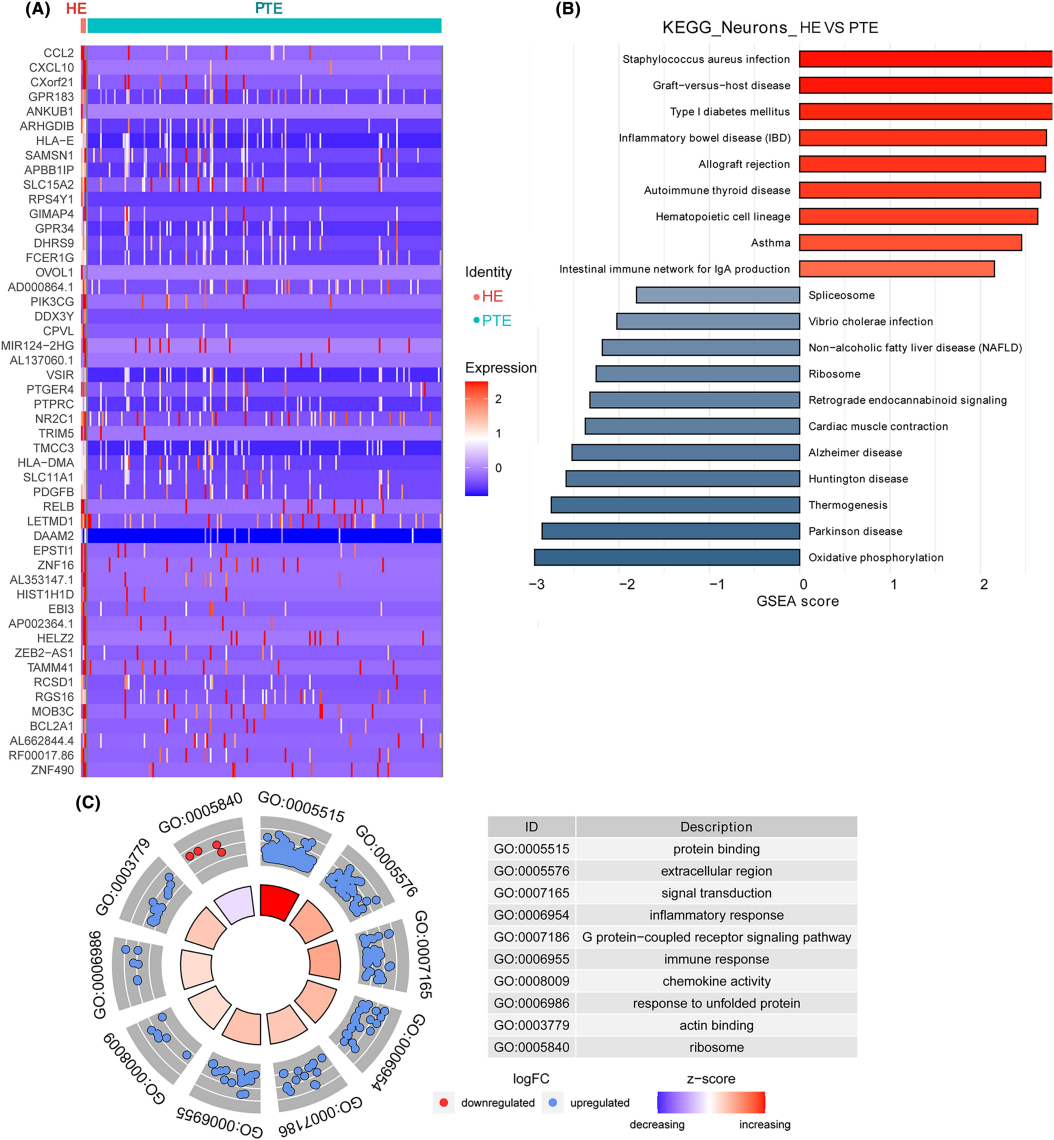
DEGs identification and function analysis in neurons. (A) A heatmap of DEGs in neurons. (B) The KEGG enrichment of DEGs in neurons. (C) A GO analysis of DEGs in neurons. DEGs, differentially expressed genes; GO, Gene Ontology; KEGG, Kyoto Encyclopedia of Genes and Genomes.

A total of 393 DEGs were identified in neurons, with 305 being upregulated and 88 downregulated (Appendix S5; Figure 6A). The most upregulated DEGs were *CCL2*, *CXCL10*, *CCL4*, *SPP1*, and *CCL4L2*; the most downregulated DEGs were *XIST*, *MTND1P23*, growth arrest and DNA damage protein 45A (*GADD45A*), posttranslational modifications (*PTMS*), and metallothionein III (*MT3*) (Figure S4).

The top 10 KEGG pathways enriched in the DEGs of astrocytes were Thermogenesis, Huntington's disease, Ribosome, Alzheimer's disease, Parkinson's disease, Spliceosome, Oxidative phosphorylation, Non‐alcoholic fatty liver disease (NAFLD), Retrograde endocannabinoid signaling, and Cardiac muscle contraction (Figure 6B).

The top 10 GO terms identified by DEGs in neurons were protein binding, extracellular region, signal transduction, inflammatory response, G protein‐coupled receptor signaling pathway, immune response, chemokine activity, response to unfolded protein, actin binding, and ribosome (Figure 6C).

## 4 DISCUSSION

Epilepsy is a chronic recurrent transient brain dysfunction syndrome characterized by recurrent epileptic seizures, and with a high prevalence second to only that of stroke. Previous studies have focused on the molecular characterization of multiple homeostatic functions in tissue samples or cell populations, in which biological differences between cells can be obscured by averaging or being mistaken for technical noise.^8^, ^17^ However, the cell types in the same tissue can vary, resulting in unique dysregulated genes and functions. With the development of scRNA‐Seq technology, single‐cell transcriptomics has become increasingly used for analyzing the cellular architecture of complex tissues and for classifying cells according to specific criteria. The novelty of our study is that we identified differences in the cell grouping signatures between post‐traumatic and hereditary epilepsy patients, and the transcriptome in different cells.

In the present study, we classified the cell types present in post‐traumatic and hereditary epilepsy and found that the DEGs and their functions in different cell types varied. After separating the cells by marker genes, the cells in brain tissues were grouped into categories of oligodendrocytes, microglial cells, OPCs, endothelial cells, smooth muscle cells, neurons, astrocytes, T cells, and progenitors The results of our present study were consistent with those reported by Oldham et al.,^18^ in which their sequencing data generated a list of co‐expressed genes that corresponded to neurons, oligodendrocytes, astrocytes, and microglia cells. Furthermore, the cell types in humans were similar to those in other vertebrate brains.^19^ The neuron is the basic unit in the brain and nervous system and is responsible for transmitting neruo‐signals. Both PTE and HE are strongly associated with neurons.^20^ In PTE, the brain may cause the abnormal excitement and discharge of neurons after the brain is traumatized or injured, resulting in seizures. This abnormal neuron activity can cause symptoms of epilepsy, such as convulsions, loss of consciousness, and emotional fluctuations. Therefore, neurons play an important role in PTE. Studies of neuron function and activity may help to understand and treat PTE. Moreover, genetic mutations might also be a key factor for HE. In our present study, the numbers and percentages of neurons were relatively low. This situation might have been caused by characteristics of the sample, as neurons are difficult to dissolve. This might have resulted in inadequate collection during the process of dismissing single cells. In addition, surgery might be another factor that influenced the samples. Although the numbers of neurons were relatively low, the numbers of DEGs identified in neurons were more than those in identified in oligodendrocytes, microglia, and astrocytes, showing an active function of neurons in PTE and HE. Therefore, we believe that the number of neurons had little influence on the DEGs.

Higher numbers of oligodendrocytes and astrocytes and lower numbers microglia cells and neurons were observed in HE than in PTE. Glial cells of astrocytes and microglia have been intensely studied in epilepsy, and results revealed an association between glial function and functional imbalance of excitatory versus inhibitory synapses.^21^ Experiments have proven that epilepsy is closely related to concentrations of extracellular glutamate.^22^ Dysfunction of the glutamate pathway may also be one of the causes of intractable epilepsy. Although extracellular glutamate only comes from neurons, astrocytic keratinocytes play a critical role in maintaining extracellular glutamate homeostasis.^23^ First, astrocytes can release glutamate. Second, approximately 80%–90% of extracellular glutamate is taken up and cleared by astrocyte glutamate transporters. Moreover, astrocytes also affect the balance of water and potassium ion metabolism.^24^ Previous studies have shown that anything that impairs the uptake of potassium by glial cells can promote convulsions.^25^, ^26^ Hippocampal slice experiments conducted in patients with refractory temporal lobe epilepsy and hippocampal sclerosis have confirmed that high potassium levels can induce epileptiform activity. Therefore, a decreased number of astrocytes is one essential iconic feature of PTE.

Microglia cells have been shown to mediate inflammation, neuronal death, and aberrant neurogenesis after an epileptic seizure.^27^ Moreover, microglia are brain‐resident immune cells that patrol the brain and control synaptic numbers either by pruning synapses or promoting synapse formation.^28^ We found that the number of microglia cells was decreased in HE when compared with PTE (2242 vs. 3811). The scRNA‐seq of non‐affected brain areas of patients with epilepsy revealed 10 distinct microglial clusters, with the genes *CCL2*, *CCL4*, and *SPP1* being involved.^14^ A similar study was conducted by Sankowski et al.,^15^ in which *CCL2* and *SPP1* were found to be dysregulated. In our present study, the genes *CCL2*, *CXCL10*, and *SPP1* expression were found in neurons in PTE compared with HE. We also found that the neurons in HE were almost absent when compared with PTE (3 vs. 203). In addition, a GO analysis indicated that the DEGs in neurons participated in inflammatory and immune responses, with the *CCL2* and *CCL4* inflammation‐related genes being involved.

Our data showed that the DEGs in microglia cells and astrocytes were involved in the IL‐17 signaling pathway. Kumar et al.^29^ revealed the pro‐inflammatory effect of IL‐17 pathways which dominate the architecture of the immunosome in pediatric refractory epilepsy. Therefore, we conclude that the DEGs in microglia cells and astrocytes are mainly involved in proinflammatory functions, and the IL‐17 signaling pathway can be considered as a treatment target and biomarker for PTE. Taken together, investigations of inflammatory and immune functions might be the best approach for studying PTE in neurons.

Only three DEGs were commonly expressed in the four cell types; these included *MTND1P23* (downregulated), *XIST* (downregulated), and *RPS4Y1* (upregulated). In a recent study, *MTND1P23* was reported to be dysregulated in the lateral temporal lobe of the brain fusiform gyrus,^30^ revealing its activation in the lateral temporal lobe. However, studies on the role of *MTND1P23* in epilepsy remain inadequate. *XIST* was found to be dysregulated in spinal cord trauma and reported to be involved in the biological processes of inflammation, oxidation, and apoptosis.^31^, ^32^ Moreover, *XIST* also increases inflammatory cell infiltration and fibrosis,^33^ promotes satellite glial cell activation, and aggravates inflammatory pain.^34^ When compared with PTE, the level of *XIST* expression was significantly downregulated in HE, revealing considerable differences in terms of inflammation between traumatic epilepsy and hereditary epilepsy. Moreover, *XIST* plays an essential role in PTE.

## 5 CONCLUSION

In conclusion, our study explored differences in cell groupings between traumatic and hereditary epilepsy as well as the transcriptome in different cells. We found that higher numbers of oligodendrocytes and astrocytes, and lower numbers of microglia cells and neurons were present in HE than in PTE. The IL‐17 signaling pathway might be a treatment target and biomarker for PTE in microglia cells and astrocytes. Studying inflammatory and immune functions might be the best approach for investigating PTE in neurons. *XIST* is a common DEG that plays an important role in different cells.

## AUTHOR CONTRIBUTIONS

Study design/planning, data analysis/statistics, and data interpretation: JX and FW. Data collection/entry: FW and ZT. Preparation of manuscript: FW and JX. Literature analysis/search: FW and DH. Funds collection: JX.

## CONFLICT OF INTEREST STATEMENT

The authors declare that the research was conducted in the absence of any commercial or financial relationships that could be construed as a potential conflict of interest.

## CONSENT TO PARTICIPATE

The two patients in this study provided their written informed consent.

## Supporting information


Figures S1–S4

Table S1

Table S2

Table S3

Appendices S1–S5

## Data Availability

The datasets generated during and/or analyzed during the current study are available from the corresponding author on reasonable request.
