# Supplementary material for: Molecular mechanism analyses of post‐traumatic epilepsy and hereditary epilepsy based on 10× single‐cell transcriptome sequencing technology
Source: CNS Neurosci Ther. 2024 Apr 4;30(4):e14702. doi: 10.1111/cns.14702 (PMC10993349; doi:10.1111/cns.14702)
Supplement: Supplementary file 1 — Figures S1–S4 [file CNS-30-e14702-s002.zip › FiguresS1-S4-Legends.docx]

**Figure S1** The top 5 differentially expressed genes in PTE-oligodendrocytes and HE-oligodendrocytes.

**Figure S2** The top 5 differentially expressed genes in PTE-microglial cells and HE-microglial cells.

**Figure S3** The top 5 differentially expressed genes in PTE-astrocytes and HE-astrocytes.

**Figure S4** The top 5 differentially expressed genes in PTE-neurons and HE-neurons.
