# Supplementary figures and images for: Molecular mechanism analyses of post‐traumatic epilepsy and hereditary epilepsy based on 10× single‐cell transcriptome sequencing technology
Source: CNS Neurosci Ther. 2024 Apr 4;30(4):e14702. doi: 10.1111/cns.14702 (PMC10993349; doi:10.1111/cns.14702)

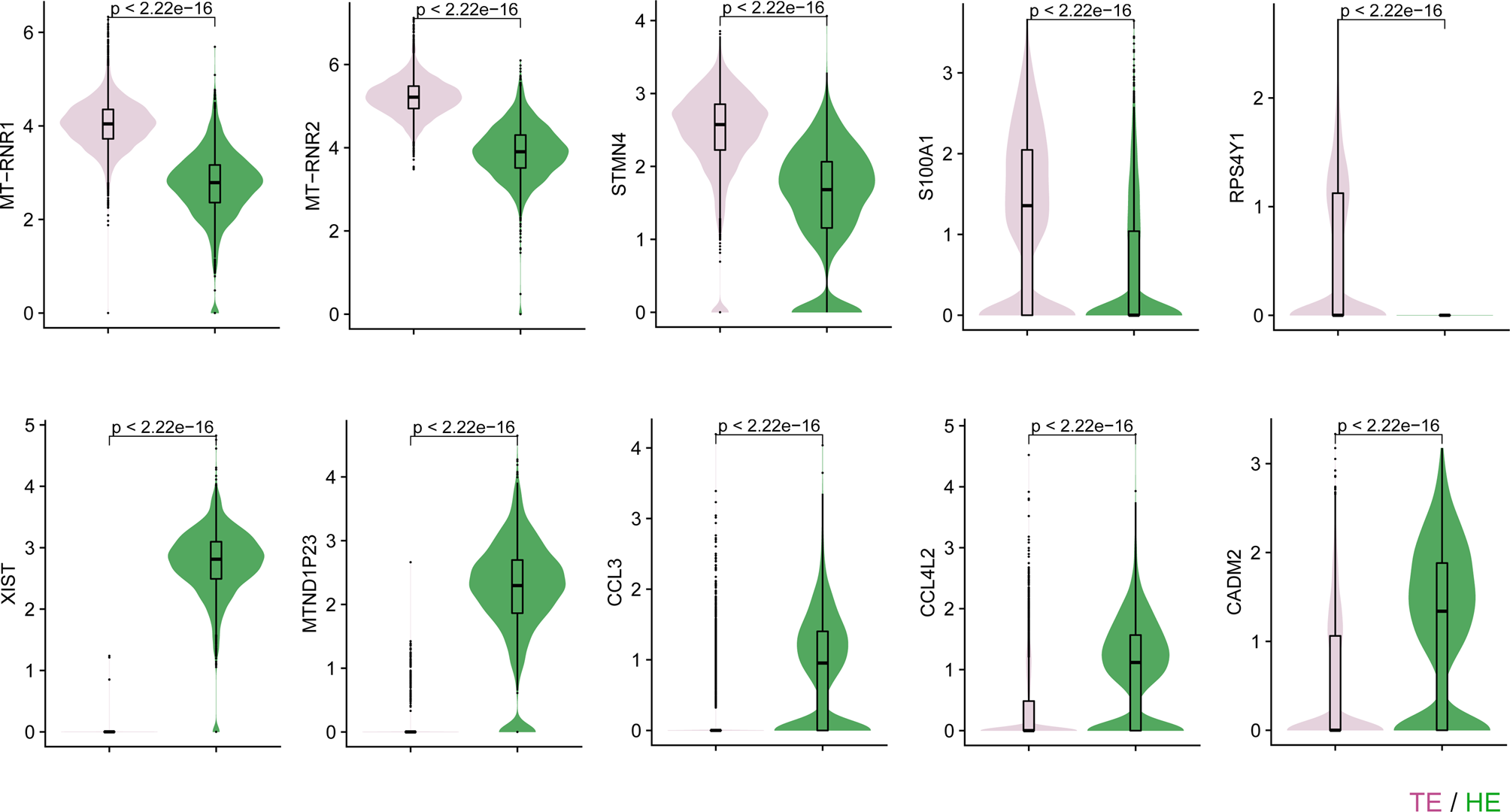

Supplement: Supplementary file 1 — Figures S1–S4 [file CNS-30-e14702-s002.zip › FigureS1.tif]

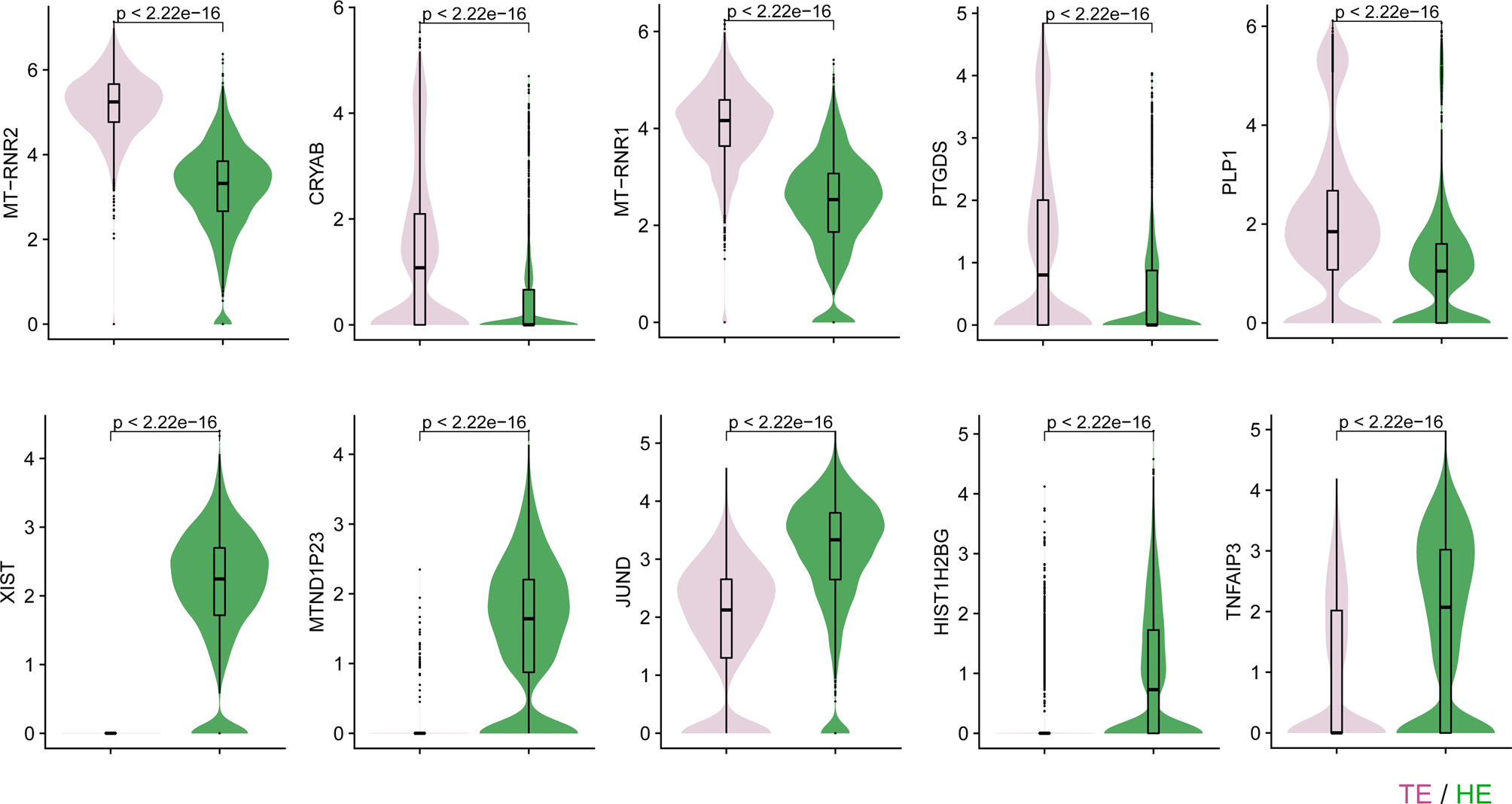

Supplement: Supplementary file 1 — Figures S1–S4 [file CNS-30-e14702-s002.zip › FigureS2.tif]

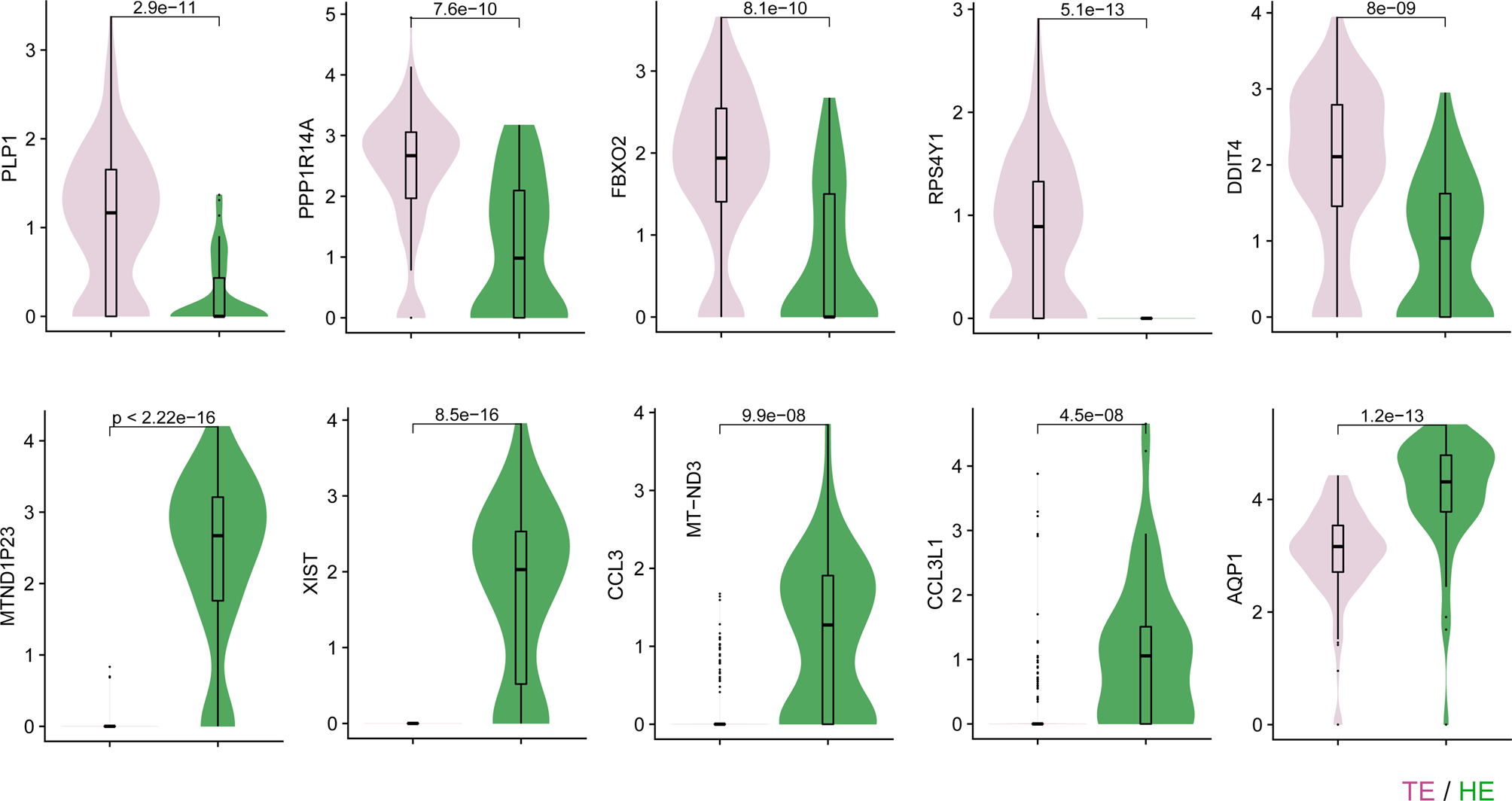

Supplement: Supplementary file 1 — Figures S1–S4 [file CNS-30-e14702-s002.zip › FigureS3.tif]

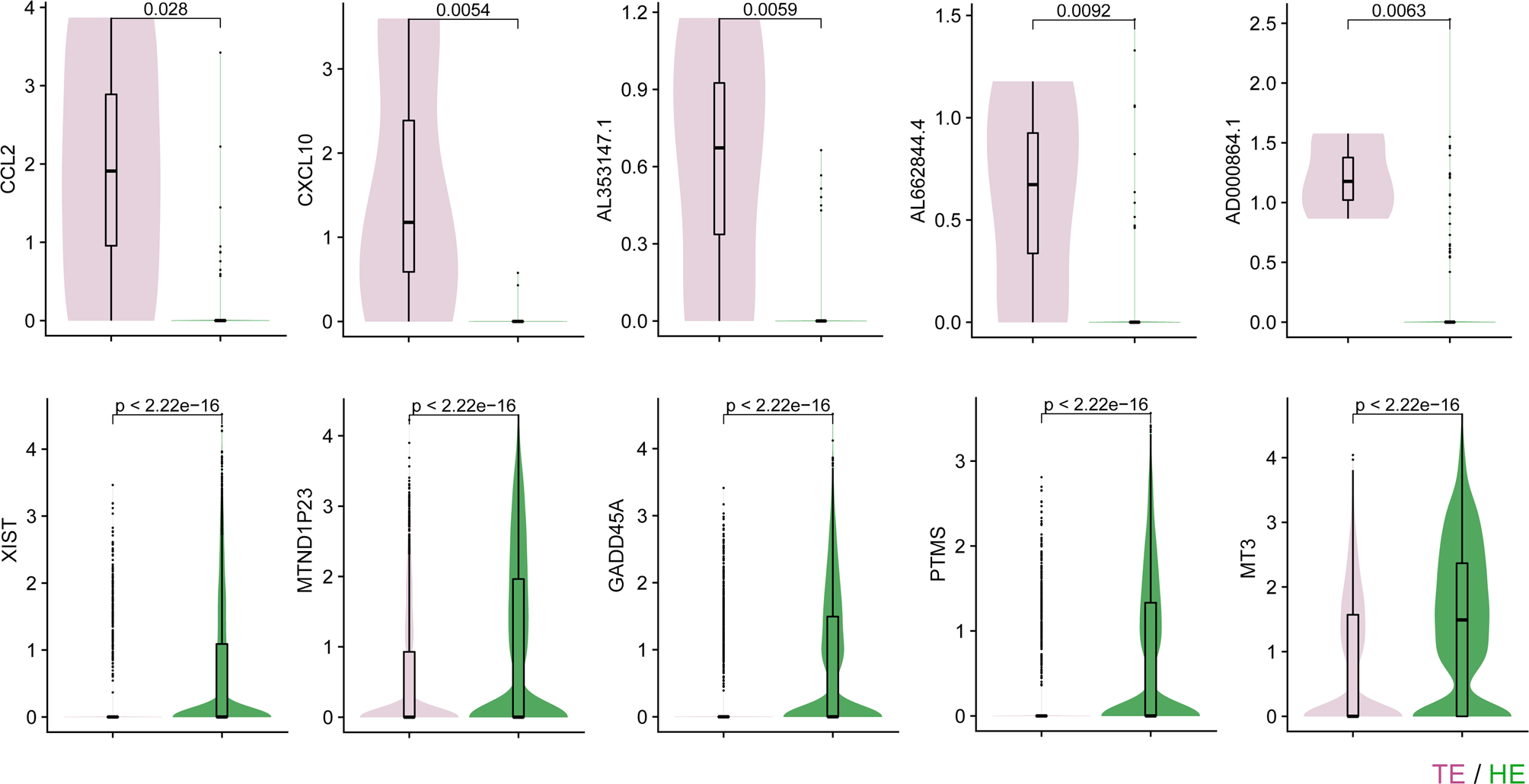

Supplement: Supplementary file 1 — Figures S1–S4 [file CNS-30-e14702-s002.zip › FigureS4.tif]
