# Supplementary material for: Molecular mechanism analyses of post‐traumatic epilepsy and hereditary epilepsy based on 10× single‐cell transcriptome sequencing technology
Source: CNS Neurosci Ther. 2024 Apr 4;30(4):e14702. doi: 10.1111/cns.14702 (PMC10993349; doi:10.1111/cns.14702)
Supplement: Supplementary file 2 — Table S1 [file CNS-30-e14702-s005.docx]

**Figure S1** The top 5 differentially expressed genes in PTE-oligodendrocytes and HE-oligodendrocytes.

**Figure S2** The top 5 differentially expressed genes in PTE-microglial cells and HE-microglial cells.

**Figure S3** The top 5 differentially expressed genes in PTE-astrocytes and HE-astrocytes.

**Figure S4** The top 5 differentially expressed genes in PTE-neurons and HE-neurons.

**Table S1. The basic characteristic of epilepsy patients.**

| **Characteristics** | **Traumatic epilepsy patient** | **Hereditary epilepsy patient** |
| --- | --- | --- |
| Gender | Male | Male |
| Age (year) | 18 | 15 |
| Date of first occurrence | 16 years old | 3 years old |
| Description of first occurrence | After the patient was injured in a car accident two years ago, he suddenly twitched his limbs, foamed at the mouth, became delirious, and couldn't call, lasting for about 3 minutes. Then craniotomy exploration and hematoma removal were finished. | When the patient was 3 years old, the first attack was characterized by limb convulsions, foaming at the mouth, eyes upturned, and unconsciousness for about half an hour. |
| Medical history | Sodium valproate (200mg bid) + Oxcarbazepine (300mg bid) | Lamotrigine (75mg bid) |
| Frequency of recent occurrence | About once 1 month | About once every 10 days |
| Diagnosis | 1. Traumatic epilepsy; 2 Right frontotemporal lobe malacia; 3. Right hippocampal sclerosis | 1. Epilepsy; 2. Dysplasia of right temporal cortex; 3. Right hippocampal sclerosis |
| Operation | Epilepsy knife; Resection of right frontotemporal softened foci + right medial temporal structures. | Epilepsy knife; Resection of right anterior temporal lobe + right medial temporal lobe. |
