# Supplementary material for: Molecular mechanism analyses of post‐traumatic epilepsy and hereditary epilepsy based on 10× single‐cell transcriptome sequencing technology
Source: CNS Neurosci Ther. 2024 Apr 4;30(4):e14702. doi: 10.1111/cns.14702 (PMC10993349; doi:10.1111/cns.14702)
Supplement: Supplementary file 3 — Table S2 [file CNS-30-e14702-s004.docx]

**Table S2. The number and percentage of cells distributed in two groups**

| **Clusters** | **HE Number** | **HE Percentage (%)** |  | **PTE Number** | **PTE Percentage (%)** |  |
| --- | --- | --- | --- | --- | --- | --- |
| Oligodendrocytes | 6522 | 69.70 |  | 2532 | 33.85 |  |
| Microglia | 2242 | 23.96 |  | 3811 | 50.96 |  |
| OPCs | 232 | 2.48 |  | 298 | 3.98 |  |
| Endothelial cell | 92 | 0.98 |  | 249 | 3.33 |  |
| Smooth muscle cell | 24 | 0.26 |  | 232 | 3.10 |  |
| Neurons | 3 | 0.03 |  | 203 | 2.71 |  |
| Astrocytes | 133 | 1.42 |  | 52 | 0.70 |  |
| T cell | 92 | 0.98 |  | 76 | 1.02 |  |
| Progenitors | 17 | 0.18 |  | 26 | 0.35 |  |
