# Supplementary material for: Molecular mechanism analyses of post‐traumatic epilepsy and hereditary epilepsy based on 10× single‐cell transcriptome sequencing technology
Source: CNS Neurosci Ther. 2024 Apr 4;30(4):e14702. doi: 10.1111/cns.14702 (PMC10993349; doi:10.1111/cns.14702)
Supplement: Supplementary file 4 — Table S3 [file CNS-30-e14702-s001.docx]

**Table S3. The number of DEGs in four cell clusters**

| Cell clusters | All DEGs | Upregulated DEGs | Downregulated DEGs |
| --- | --- | --- | --- |
| Oligodendrocyte | 25 | 13 | 12 |
| Microglia | 87 | 42 | 45 |
| Astrocytes | 222 | 115 | 107 |
| Neurons | 393 | 305 | 88 |

Note: DEGs, differentially expressed genes.
